# Design and Application of a High-G Piezoresistive Acceleration Sensor for High-Impact Application

**DOI:** 10.3390/mi9060266

**Published:** 2018-05-28

**Authors:** Xiaodong Hu, Piotr Mackowiak, Manuel Bäuscher, Oswin Ehrmann, Klaus-Dieter Lang, Martin Schneider-Ramelow, Stefan Linke, Ha-Duong Ngo

**Affiliations:** 1 Department Electrical Engineering, Technische Universität Berlin, Gustav-Meyer-Allee 25, 13355 Berlin, Germany; Manuel.Baeuscher@izm.fraunhofer.de (M.B.); Oswin.Ehrmann@izm.fraunhofer.de (O.E.); kdlang@izm.fraunhofer.de (K.-D.L.); 2 Wafer Level System Integration, Fraunhofer Institute for Reliability and Microintegration, Gustav-Meyer-Allee 25, 13355 Berlin, Germany; Piotr.Mackowiak@izm.fraunhofer.de (P.M.); martin.schneider-ramelow@izm.fraunhofer.de (M.S.-R.); 3 Department Development, TE Connectivity GmbH, Hauert 13, 44227 Dortmund, Germany; Stephan.Linke@te.com; 4 Microsystems Engineering, University of Applied Sciences Berlin, Wilhelminenhofstraße 75A, 12459 Berlin, Germany

**Keywords:** high acceleration sensor, piezoresistive effect, MEMS, micro machining

## Abstract

In this paper, we present our work developing a family of silicon-on-insulator (SOI)–based high-g micro-electro-mechanical systems (MEMS) piezoresistive sensors for measurement of accelerations up to 60,000 g. This paper presents the design, simulation, and manufacturing stages. The high-acceleration sensor is realized with one double-clamped beam carrying one transversal and one longitudinal piezoresistor on each end of the beam. The four piezoresistors are connected to a Wheatstone bridge. The piezoresistors are defined to 4400 Ω, which results in a width-to-depth geometry of the pn-junction of 14 μm × 1.8 μm. A finite element method (FEM) simulation model is used to determine the beam length, which complies with the resonance frequency and sensitivity. The geometry of the realized high-g sensor element is 3 × 2 × 1 mm^3^. To demonstrate the performance of the sensor, a shock wave bar is used to test the sensor, and a Polytec vibrometer is used as an acceleration reference. The sensor wave form tracks the laser signal very well up to 60,000 g. The sensor can be utilized in aerospace applications or in the control and detection of impact levels.

## 1. Introduction

Nowadays, high-g sensors have become an important measurement unit in technological applications. The areas where sensors are most commonly applied include aerospace technologies, military and security systems, and renewable energy technologies [1,2,3]. It is important to reduce the size of the sensor to extend the field of applications. A silicon microfabrication technique makes it possible to reduce both the size of the sensor and the production cost through batch fabrication, making it suitable for mass production. In state-of-the-art technology and research, high-g acceleration sensors measure the acceleration in one- or three-axis with proof masses [3,4,5,6,7]. Therefore, the electrical variation from stress influence is an important parameter. Another important aspect, relevant to the accelerometer sensitivity, is the maximum displacement of the system. Obtaining a high sensitivity is the goal of recent research. Hence, new sensing mechanisms, like silicon nanowires, will be developed. These mechanisms, with their new materials, are difficult to manufacture and not yet economically-feasible. For this reason, research and development still focuses on geometry and design optimization [8]. Therefore, a novel high-g sensor with double-clamped beam was developed at Fraunhofer IZM with a measurement range of up to 60,000 g. This paper describes the concept, the simulation, and the process flow of the sensor, with device characterization at the end. For this high-g sensor, the piezoresistive effect is used. It is a stable and well-known state-of-the-art method, with a simple evaluation unit and precise accuracy. Important aspects of this developed sensor are its high robustness and its resolution. The production process of this sensor family aims to be precise and low-cost to fulfill economic requirements and make it accessible for a variety of new applications.

## 2. Sensor Design

**Figure 1:**
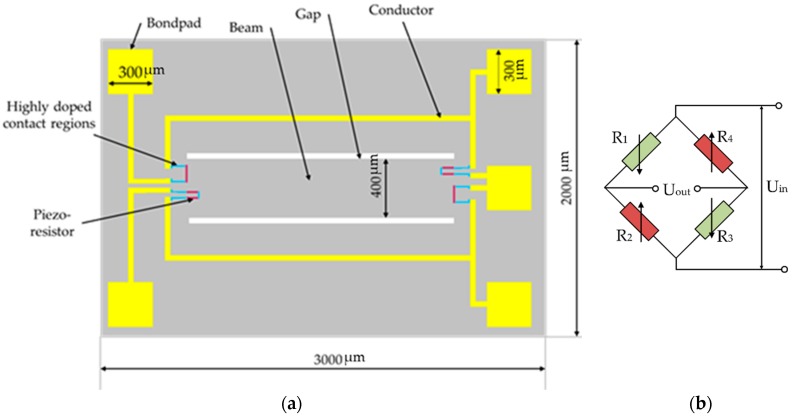
(**a**) Sketch of top view of sensor design, featuring one double-clamped beam carrying one transversal and one longitudinal piezoresistor on each end of the beam; (**b**) the Wheatstone bridge for equivalent circuit.

The developed sensor design contains a silicon beam with four integrated piezoresistors. The geometry of the sensor is optimized for a high acceleration range up to 60,000 g. Figure 1 shows a sketch of the top view of the sensor design.

Piezoresistors and conductors are connected to form an open full Wheatstone bridge. It contains one double-clamped beam and carries one transversal and one longitudinal piezoresistor on each end of the beam. Highly doped contact regions are employed to connect the piezoresistors to conductors. The overall chip size is 3 mm × 2 mm in length and width, respectively. According to Equation (1), a change of mechanical strain on the Wheatstone bridge is transformed into a change of output voltage of the piezoresistive acceleration sensor.

(1) $$\Delta U=U_{0}\cdot \varepsilon \cdot k$$

where Δ*U* represents the change of the output voltage and *U*_0_ the supply voltage of the Wheatstone bridge. To increase the sensitivity, both the piezoresistive gauge factor *k* and the mechanical strain *ɛ* can be increased. 

The gauge factor of silicon depends on the dopant concentration and is typically limited to values below 100. Piezoresistors with a high gauge factor are also more sensitive to changes of temperature, i.e., they have a higher temperature coefficient of resistance (TCR). To obtain a high sensitivity, a dopant concentration of 2 × 10^18^/cm^3^ after annealing is implanted to form the piezoresistors, which should have a gauge factor of at least 90. The high influence of temperature on such piezoresistors is accepted to obtain a very high sensitivity without coming close to the yield strain of silicon. For the design of the dimensions of the piezoresistors, the joule heating effect should also be considered, which is one of the biggest influencing factors for the resistors’ performance. For this reason, the current density within each resistor should be kept low. The increase of electrical resistance and of the piezoresistors’ cross-section helps to reduce the current density. Therefore, the values of resistance are defined as 4400 Ω and the cross section of the piezoresistors is designed as 14 μm × 1.8 μm (width × depth of pn-junction, respectively).

(2) $$\varepsilon =\frac{\rho \cdot f\cdot g}{2\cdot E\cdot t}\cdot l^{2}$$

The mechanical strain can be calculated with Equation (2), which is based on the beam theory for a double-clamped beam. Where the density *ρ* and Young’s modulus *E* are fixed material parameters, and load *f* and gravity *g* are determined by external factors, only the geometrical parameters of beam thickness *t* and of beam length *l* can be modified to increase the strain. To provide high sensitivity as well as sufficient mechanical strength to tolerate a maximal acceleration overload of twice the specified full range acceleration, the beam thickness is set to 20 μm [2,9]. Therefore, the only mechanical design parameter changeable to provide a higher strain and thus a higher sensitivity is the beam length *l*. The FEM model can be used to determine the beam length, which complies with the resonance frequency and sensitivity. While the sensitivity of the sensor is increased by increasing the beam length, its resonance frequency decreases (stability). Sensor design is therefore always a compromise between high sensitivity and high resonance frequency. Another parameter that should be considered is the beam width, because all four of the piezoresistors should be placed on the beam ends to form a full Wheatstone bridge configuration within the areas of high strain on the beam ends. However, according to Equation (2), the beam width has no influence on the strain the piezoresistors are subjected to. In this project, a beam with a width of 400 μm was set for the double-clamped beam. 

**Figure 2:**
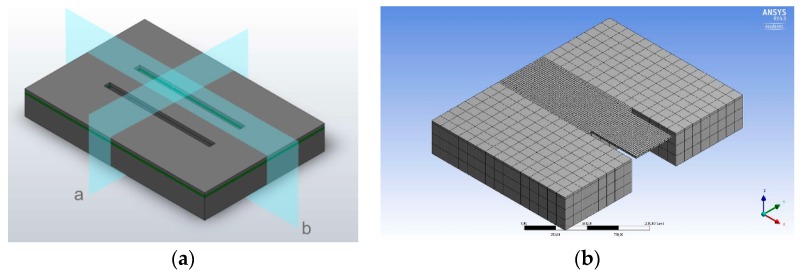
(**a**) Symmetrical axis of acceleration sensor; (**b**) mashed model half of used sensor. Meshing is homogenous over the surface with element sizes of 20 µm in the beam area and 100 µm around the beam area on the bulk material.

To analyze the mechanical behavior of the novel double clamped beam structure and to determine the length of the beam length, a simplified model was built, as shown in Figure 2. Here, a quarter of the model is shown, for symmetry of the sensor design can be exploited the reduce computational time. The influence of the different beam lengths on the resonance frequency and sensitivity of piezoresistive sensors were calculated and simulated. A static mechanical analysis using ANSYS mechanical was generated. The element type used was Solid64, as it defines eight knots and three degrees of freedom. As border conditions for this simulation, the surface of the sensor was defined free of displacement. The sensor’s symmetry was exploited, and therefore, the symmetrical borders were set free of displacement in the normal direction of symmetry.

The resonance frequency is a direct result of the modal analysis carried out for each beam configuration. Equation (3) can be used to estimate the first mode resonance frequency of a double-clamped beam.

(3) $$f_{1}=\frac{\frac{e_{1}{}^{2}\cdot t}{l^{2}}\sqrt{\frac{E}{12\cdot \rho }}}{2\cdot \Pi }$$

where the first resonance frequency *f*_1_, beam length *l*, Density *ρ*, and Young’s modulus *E*. Among them, *ρ* and *E* are fixed material parameters, and *f*_1_ will decrease with increasing *l*. The influence of the different beam lengths on the sensitivity of piezoresistive sensors to acceleration *S_a_* is calculated by Equation (4).

(4) $$S_{a}=\frac{\Delta U}{\Delta a\cdot U_{0}}$$

**Figure 3:**
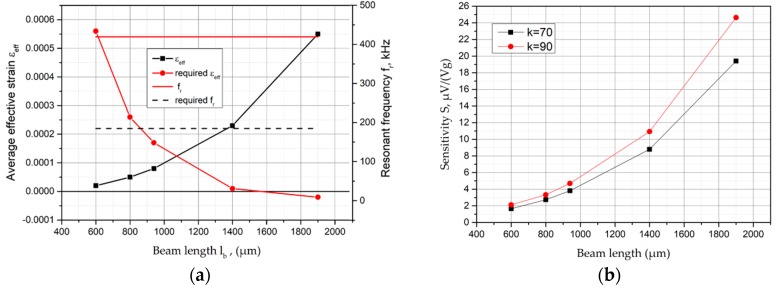
(**a**) Simulated average effective strain and resonance frequency over beam length at an acceleration load of 60,000 g; (**b**) sensitivity at full scale, calculated from the simulated average effective strain with an SOI thickness of 20 µm and *k*-factors of 70 and 90, respectively.

Using a gauge factor of 90, the required strain to obtain the specified sensitivity can be calculated. By extracting the strain values for each beam configuration at the location of the piezoresistors from the simulation result files, the average strain of the piezoresistors were evaluated. The simulation values for resonance frequency and strain were plotted over the beam length for the acceleration ranges of 60,000 g in Figure 3a, and sensitivity at full scale, calculated from the simulated average effective strain with SOI thickness of 20 µm and *k*-factors of 70 and 90, were plotted in Figure 3b.

The simulated values given in Figure 3a were divided by the required values for resonance frequency and strain. The intersection points of required and simulated strain and frequency allow the reduction of the beam length, complying with the given specifications. At an acceleration of 60,000 g, a beam of not more than 600 μm length will comply with the frequency specification. To obtain the required strain, a beam length of more than 14,000 μm is required at 60,000 g. Based on the simulation results presented here, the selection of specific sensor geometries, fulfilling at least one of the sensor specifications, is possible. The simulations clearly show that no single sensor design exists to fulfill both the resonance frequency and sensitivity specification for the acceleration range of 60,000 g. Thus, the establishment of a beam length is a compromise between resonance frequency and average effective strain.

## 3. Fabrication Flow of the Sensor Wafer

The sensors were produced from SOI wafers with a device layer of 20 μm. Table 1 lists the thickness of each layer during the sensor process. A handle-wafer thickness from 300 μm to 325 μm, a buried-oxide thickness from 0.2 μm to 0.4 μm, and a base dopant concentration of approximately 1 × 10^15^ phosphorus ions/cm^3^ are recommended.

**Figure 4:**
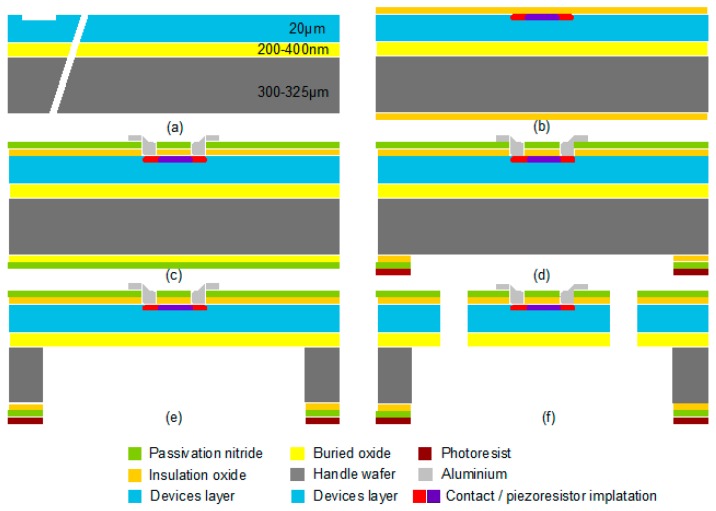
Fabrication flow of a high-g acceleration sensor. (**a**) Cross-section of SOI wafer after dry etching of the alignment marks; (**b**) after contact and piezoresistor implantation and photoresist removal; (**c**) after structuring of the metallization layer. (**d**) After structuring of the backside nitride and oxide layer; (**e**) after etching of the handling wafer from the back side; (**f**) after release of the beam structure by wet etching.

The fabrication process of the sensor, which is based on the micromachine technology, is shown in Figure 4. The first processing step was aimed at etching the alignment marks into the device layer of the wafers (see Figure 4a). For this purpose, a standard photolithographic process flow composed of photoresist spin coating, prebake, exposure, development, and postbake was implemented. The second step was to implant the contact region and the piezoresistors. Before the implantation, a layer of approximately 50-nm-thick silicon oxide was grown in a furnace at 1000 °C (stray oxide). Then the implantation of contact regions and piezoresistors was accomplished with the standard photolithographic process described above. A dopant dose of 3 × 10^15^ and one of 1.1 × 10^14^ boron ions/cm^2^ were used for the contact regions and piezoresistors, respectively. An implantation energy of 60 keV and a maximum beam current of 100 μA were used for the implantation. After the implantation, the wafers were cleaned and prepared for the annealing and oxidation process. Annealing and oxidation took place simultaneously in a furnace at 1000 °C. Furthermore, SUPREM simulations indicated that an annealing time of eight hours was necessary to establish the ion concentration of approximately 2 × 10^18^/cm^3^ required to obtain a gauge factor of 90 and to realize a pn-junction depth of 1.8 μm. At the beginning of the annealing, oxygen was added to the furnace gas to grow a layer of insulating silicon oxide. After the annealing and oxidation, a silicon nitride layer was deposited through a low-pressure chemical vapor deposition (CVD) process. The next step was to establish electrical contact between the metallization layer and the contact regions. In order to form conductors and bond pads, the standard photolithographic process and dry etching process were employed to open the contact areas. As demonstrated in Figure 4c, a thin AlSiCu layer was then sputtered onto the surface and into the contact holes of the wafer. After structuring the AlSiCu layer by chemical wet etching and removing the photoresist, the metal layer was annealed in a forming gas atmosphere at 450 °C to establish ohmic contact between the metallization layer and the implanted contact regions.

**Figure 5:**
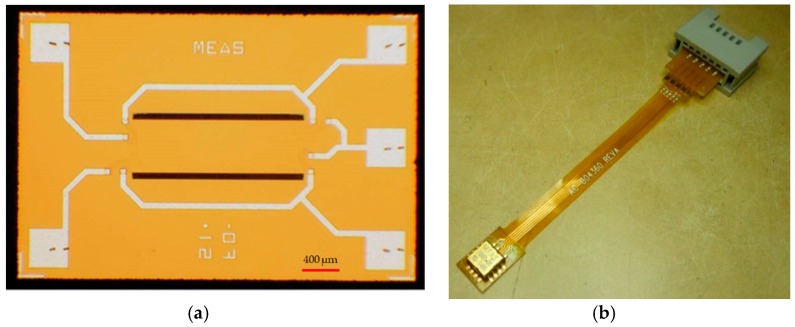
(**a**) A realized MEMS high-g-sensor element (3 × 2 × 1 mm^3^). On top, the silicon beam with integrated piezoresistors, the leads, and the bond pads can be clearly seen; (**b**) packaged high-g-sensor system.

To realize the mechanical sensor structure, the process started by coating the back side of the wafer with a thick photoresist layer (see Figure 4d). This layer served as an etch mask for deep silicon etching, and therefore it is typically spun at a low rotational speed to obtain a thickness of over 4 μm. The photoresist edge bead was removed with acetone on a spin coater after the prebake to ensure good wafer clamping during the deep silicon etching. After exposure, development, and postbake, the back side photoresist mask featured openings for the back side cavities. Before deep silicon etching was conducted, the passivation and insulation layers needed to be removed locally. Removal of the silicon nitride was conducted by dry etching, and wet etching with the BOE was used to remove the silicon oxide. Etching of the front side AlSiCu metallization by the BOE was prevented by applying a protective foil on the wafer front side before wet etching. The silicon oxide insulation was etched within a few minutes. After the wafer was rinsed and dried, the protective foil was removed manually. Alternatively, protection of the aluminum was possible by coating the wafer top side with photoresist. The wafer was etched from its back side with the Bosch process until the BOX layer was reached and exposed to the entire cavity bottom, as seen in Figure 4e. The photoresist mask was removed subsequently. To form a double-clamped beam, two trenches per chip needed to be etched through the device layer, where deep silicon etching was also applied (see Figure 4f). Moreover, a standard photolithographic process was utilized, and after the postbake, the nitride and the oxide layers were removed locally by dry and BOE etching, respectively. A short, deep silicon etching process was sufficient to structure the device layer down to the BOX layer. After the release of the beam structure and the removal of the photoresist, the chips were finished and tested. Figure 5 displays a realized MEMS high-g-sensor element (3 × 2 × 1 mm^3^) and its package system.

## 4. Device Characterization

**Figure 6:**
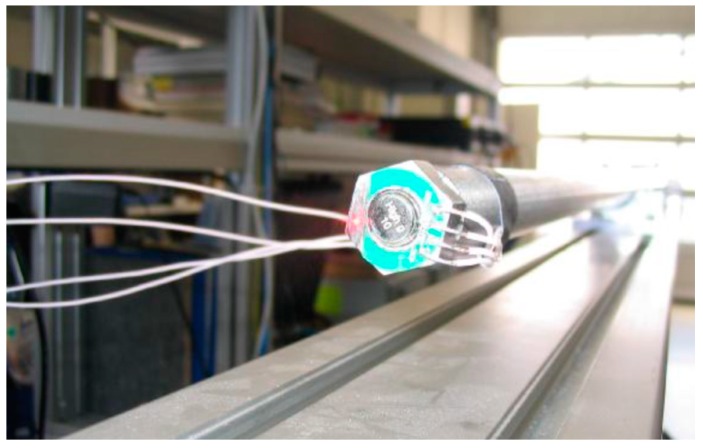
Infrastructure (Shock Wave Bar) used to characterize the sensors.

**Figure 7:**
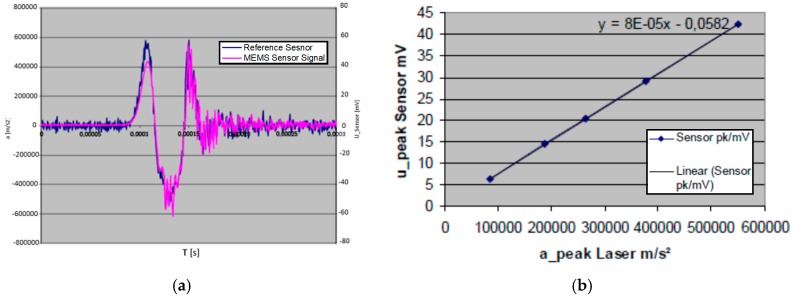
(**a**) Test results of the fabricated 60,000 g sensor. Magenta: MEMS sensor signal. Blue: reference sensor; (**b**) the calculated linearity of the sensor.

In order to generate the necessary accelerations, a shock wave bar (Figure 6) and a Polytec vibrometer were employed as an acceleration reference. The MEMS sensor element was attached to a stainless-steel test fixture using the standard die attach method. The die was wire-bonded to the Printed-Circuit-Board (PCB) where the output wires were soldered. Furthermore, a cover was added for protection. The test fixture was mounted to the end of the shock wave bar. It also served as the reference for the laser. Figure 7a shows the time domain by excitation at about 60,000 g. Note that the left scale in m/s^2^ is for the reference laser sensor, and the right scale in mV is for the tested sensor. The sensor wave form represents the laser signal. When applying higher accelerations, the time domain signal becomes more distorted because of structural ringing at the bar and test fixture interface. The linearity was also calculated from the maxima of the vibrometer and accelerometer signal. A typical output signal is demonstrated in Figure 7b. The sensor wave form tracks the laser signal very well.

## 5. Conclusions

In this paper, a novel method for a high-impact sensor with an impact up to 60,000 g is proposed. The sensor is realized with a double-clamped beam form and has been successfully fabricated using silicon micromachining and diffusion techniques. The fabricated sensor was also tested with a shock test, and the measurement results reveal that the fabricated devices exhibit a linear response. In addition, with the time domain by excitation up to 60,000 g, the sensor wave form tracks the laser signal very well. The sensors can be utilized in aerospace applications or in the control and detection of impact levels.

## Figures and Tables

**Table 1:** Thickness of each layer during the sensor fabrication.

Layers	Thickness (μm)
Handle wafer	300–325
BOX (buried oxide)	0.2–0.4
Device layer	20
Stray oxide	0.05
Insulation oxide	0.1
Silicon nitride	0.1
Pn-junction depth	1.8
